# The Effect of Different Turn Speeds on Whole-Body Coordination in Younger and Older Healthy Adults

**DOI:** 10.3390/s21082827

**Published:** 2021-04-16

**Authors:** Fuengfa Khobkhun, Mark Hollands, Jim Richards

**Affiliations:** 1 Faculty of Physical Therapy, Mahidol University, Salaya, Nakhon Pathom 73170, Thailand; fuengfa.kho@mahidol.edu; 2 Brain and Behaviour Lab, Research Institute for Sport and Exercise Sciences, Liverpool John Moores University, Liverpool L3 3AF, UK; m.a.hollands@ljmu.ac.uk; 3 Allied Health Research Unit, University of Central Lancashire, Preston PR1 2HE, UK

**Keywords:** inertial measurement unit, turning, whole-body coordination, older adults

## Abstract

Difficulty in turning is prevalent in older adults and results in postural instability and risk of falling. Despite this, the mechanisms of turning problems have yet to be fully determined, and it is unclear if different speeds directly result in altered posture and turning characteristics. The aim of this study was to identify the effects of turning speeds on whole-body coordination and to explore if these can be used to help inform fall prevention programs in older adults. Forty-two participants (21 healthy older adults and 21 younger adults) completed standing turns on level ground. Inertial Measurement Units (XSENS) were used to measure turning kinematics and stepping characteristics. Participants were randomly tasked to turn 180° at one of three speeds; fast, moderate, or slow to the left and right. Two factors mixed model analysis of variance (MM ANOVA) with post hoc pairwise comparisons were performed to assess the two groups and three turning speeds. Significant interaction effects (*p* < 0.05) were seen in; reorientation onset latency of head, pelvis, and feet, peak segmental angular separation, and stepping characteristics (step frequency and step size), which all changed with increasing turn speed. Repeated measures ANOVA revealed the main effects of speeds within the older adults group on those variables as well as the younger adults group. Our results suggest that turning speeds result in altered whole-body coordination and stepping behavior in older adults, which use the same temporospatial sequence as younger adults. However, some characteristics differ significantly, e.g., onset latency of segments, peak head velocity, step frequency, and step size. Therefore, the assessment of turning speeds elucidates the exact temporospatial differences between older and younger healthy adults and may help to determine some of the issues that the older population face during turning, and ultimately the altered whole-body coordination, which lead to falls.

## 1. Introduction

Fall-related injuries that occur while turning have been associated with an increased risk of subsequent hip fractures in elderly people [1]. Cumming and Klineberg [2] examined the association between history of falls and risk of hip fracture in 412 older adults, which identified characteristics of falls related to hip fracture. They found that individuals who fell while performing a standing turn test were 7.9 times more likely to have a subsequent fall resulting in a hip fracture. Falling in older adults can lead to immobility and loss of independence, resulting in high costs for both the individual and healthcare systems [3].

Turning is a fundamental but complex component of behavior that requires whole-body coordination, however, instability and balance impairment during turning are common in the elderly [1]. Turning is initiated by saccadic eye movements to shift gaze in the direction of travel followed by the rotation of the head, then the trunk and pelvis, and, finally, the stepping movements of the feet [4,5,6,7,8,9]. Although older adults employ the same temporal sequence as younger adults, there are significant differences in the spatial characteristics of the turn; for instance, younger adults show greater head-on-trunk rotation than older adults [10]. It has also been observed that older adults employ an en-bloc movement strategy while turning, which is characterized by reduced relative rotations between adjacent segments and a near-simultaneous rotation initiation [11].

A previous study found that staggering is common among older adults who had previously experienced an unexpected fall when completing a 180° turn [1]. Problems associated with changing direction in older adults and individuals with balance problems, particularly with the performance of 180° turns, have been reported [12]. These studies have identified similar findings with regards to stepping characteristics in older adults, with older adults reporting difficulties when performing a turning task and who were more likely to take multiple steps (three or more) to complete a 180° turn [1,12]. The stepping actions during a turn are critical as they may be an indication of instability and loss of coordination. Slow turning has been found to be associated with smaller and more frequent steps; characteristics that are also more common in older adults [13,14]. During turning in both a standing or walking turn, older adults often turn more slowly and with more rigid trunk movements, which may represent a strategy to compensate for actual or perceived instability [12,13,14]. In addition, real-life turning situations often require quick and unpredictable movements, with limited time for planning, e.g., turning to circumvent an unexpected obstacle or turn as fast as possible to get to a target. Therefore, it is important to investigate unplanned–reactive–turns incorporating speed modifications of turning patterns. To our knowledge, there are currently no studies that have investigated the effect of speed during turning in older adults, which may provide more detail regarding challenges faced by older adults when completing turns. The aims of this study were to explore the effects of the turn speed during turning over 180° on body coordination and stepping characteristics in healthy older adults when compared to healthy younger adults. We hypothesized that changing turn speed would result in changes in whole-body coordination and stepping characteristics in older adults only. This aimed to clarify the effects of turn speed on turning characteristics in older adults, which may be linked to fall risks, which in turn may be used to inform fall prevention programs in older adults.

## 2. Materials and Methods

### 2.1. Participants

Based on a previous study that used a similar methodology [15], G*Power statistical software was used to determine the sample size required by using the head onset latency variable (an effect size of f = 0.3, Alpha = 0.05, power = 0.95, sample size = 32, critical t(18) = 3.15, and Lambda = 17.28). A sample size of 16 participants per group was determined to be sufficient, however, to allow for any dropouts or missing data, the sample size was capped at least at 20 participants per group. The following inclusion criteria were considered; aged between 18–35 for the younger adults group and 60–75 for the older adults group, able to follow commands and instructions, able to walk independently without any assistive device, and have sufficient cognitive ability to understand the questionnaire and follow commands, which was assessed using the mini-Thai mental state examination with a score of ≥24/30 [16]. Participants had no clinical diagnosis of a condition or symptoms that could influence the test performance, such as arthritis or severe leg pain. The following exclusion criteria were used; comorbidity with severe systemic illness, severe signs and symptoms of musculoskeletal problems, which could influence test performance. All participants were asked to read the participant information sheet and sign an informed consent form. The study was approved by the local Ethics Committee on Human Experimentation and adhered to the Declaration of Helsinki (MU-CIRB 2020/048.1902).

### 2.2. Turning Protocol and Data Collection

**Figure 1:**
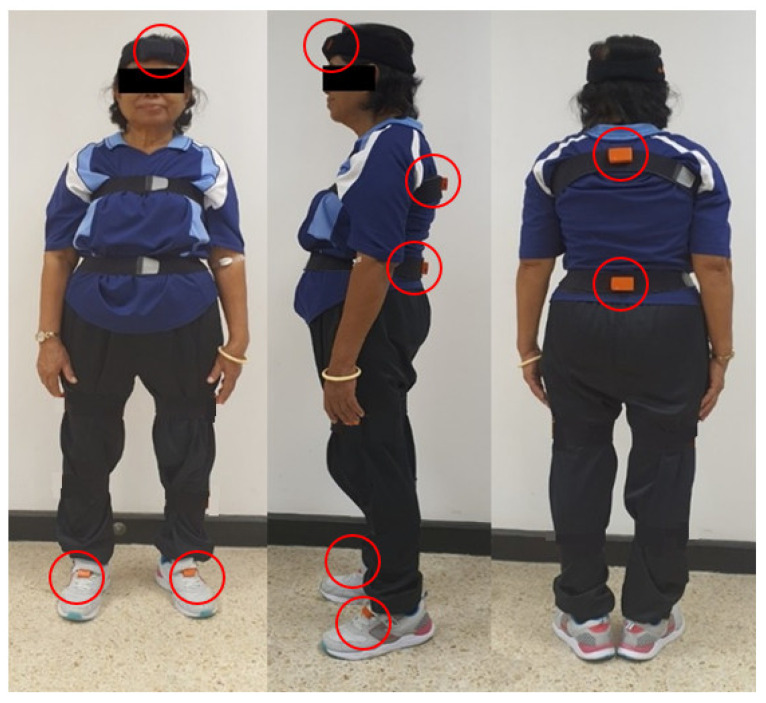
Location of the positioning of the XSENS IMU sensors.

The turning kinematics of all participants were measured using Inertial Measurement Units (XSENS, MVN, Xsens Technologies B.V., P.O. Box 559, 7500 AN Enschede, the Netherlands), which was used to measure whole-body movement turning kinematics and stepping characteristics at a sampling frequency of 100 Hz. IMUs were strapped firmly to the body segments, including the center of the head, middle of the thorax, pelvis, and the center of the left and right feet, Figure 1.

After the attachment of the XSENS sensors, the model was then calibrated for each participant before collecting data. The calibration process took approximately 10–15 s to perform and followed the manufacturer’s recommendations. This involved a static phase where the participants were asked to stand still with a relaxed upright position without moving, and a dynamic phase where the participants were asked to start walking forward with a comfortable arm swing for about 5 m and then turn to walk back to the starting position, and finally a second static phase standing still again with a relaxed upright position.

**Figure 2:**
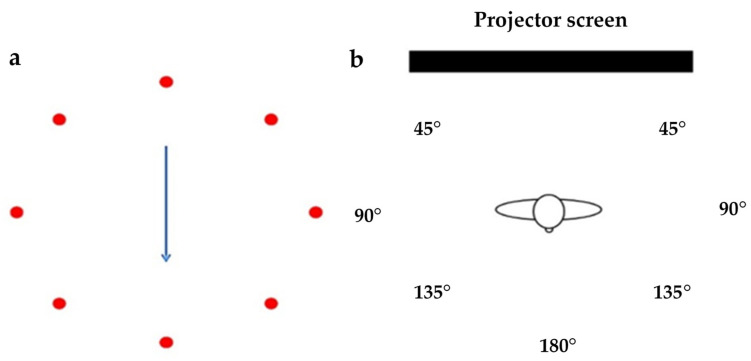
(**a**) A representation of the video screen for 180°; (**b**) participants completing standing turns on level ground through 180° in each speed from audio signals and either to the left (counter-clockwise) or the right (clockwise).

For data collection, participants stood facing a laptop screen. A test consisted of a visual cue, controlled by a program in LabVIEW, which showed a representation of the turn the participants were asked to imitate, focusing on the direction and speed of an animated clock arm as accurately as possible. Prior to each trial, a video was shown of the animation demonstrating the turn. Participants were asked to turn at 3 randomly selected speeds; fast (1.5 s), moderate (2 s), and slow (3 s), the timing of which was indicated by two audio signals, which has been used previously to explore turning speeds during 180° turns [9,17] (Figure 2). Each participant was allowed to perform a practice trial and they were instructed by the verbal instruction by “please begin turning on the first audio signal and finish turning when the audio signal finished as consistent with the audio as you can”. The instruction was also used during the test. In addition, they were asked to turn to point in a new direction, and all participants invariably ended up with their head, body, and feet aligned with the new travel direction. The test protocol was not continued until the participant indicated that they understood the instructions and the researcher was satisfied that there was no confusion about how to align their body segments. A minimum of two practice trials was performed for each direction and speed combination, and the participants were instructed to take a 5 min break at the end of the practice trials or take a rest whenever necessary until they indicated they were fully rested and ready to continue.

### 2.3. Data Processing

Segmental Euler angles from the XSENS systems were exported to determine the angular displacement of the head, thorax, pelvis, and left and right feet in the global reference frame. Kinematic data were passed through a dual fourth-order Butterworth low pass filter using a cut-off frequency of 6 Hz. The MATLAB (R2020b) programming environment was used to analyze all measures from the kinematic datasets, using the following as dependent variables; the reorientation onset time of head, trunk, pelvis and feet, peak head-trunk and peak head-pelvis angular separations, displacement, and velocity of yaw trajectory time-series from each body segment, temporal–spatial stepping characteristics including; step onset, step frequency, step duration, and step size.

To yield velocity and acceleration profiles for each segment, the displacement profiles were differentiated. The criteria used to determine the rotation onset for each segment as the earliest time point preceding segment displacement of 5° was >0° with a velocity >0°s^−1^. The end of rotation was determined as the first zero crossing in the velocity profile, following the end of the segment rotation.

Furthermore, the relationship between segmental onset latency and intersegmental kinematics was explored, which represented the intersegmental coordination during turning. The relationship between peak head yaw velocity and peak segmental angular separation has been used to indicate the extent to which the head leads the lower segments. This follows the description of previously reported techniques from these variables in clinical populations with turning deficits and has been used as a measure of coordination [11,15].

As the time-course of the turn trials varied in duration, time-normalized profiles were created for the axial segments by using the onset and offset latencies from the head and thorax. For the normalization procedure, the earliest onset latency (typically the head yaw onset latency) and the final axial offset latency were chosen for each turn trial. Normalization was performed using a customized MATLAB function, which not only increased each time series to a length of 1000 data points, i.e., longer than all individual time series but also interpolated any missing data points. This method of normalization facilitated the comparison of the segments to each other over the course of all axial segment rotations. Using the normalized axial segment profiles, angular separation profiles were obtained by subtracting one profile from another, which resulted in head-thorax and head-pelvis profiles.

Individual steps were analyzed, and the step events were defined as the positive zero crossing preceding a negative zero crossing following, a velocity that surpassed a threshold of 15% of the maximum step velocity. Each step onset was then determined as the first frame of the step interval with a velocity greater than or equal to 30°s^−1^. Following the identification of the peak velocity within an individual step, step end time was signified by the first frame being less than 30°s^−1^. Thereafter, individual step characteristics were determined from step onset to step end. Step duration, which was calculated for all steps, was defined as the interval between step onset and step placement time during the turn. The average step size was measured from the yaw rotation of the foot during the swing phase in each step while turning. The total number of steps during turning was counted from the first step to the completion of the turn. Finally, the step frequency was calculated from the number of steps taken divided by stepping duration. All dependent variables for each segment and individual stepping characteristics were extracted using a previously published methodology [15,18].

### 2.4. Statistical Analysis

All statistical analyses were performed using IBM SPSS statistics version 24 (IBM Corporation, Armonk, NY, USA). The distribution of all data was tested using Shapiro–Wilk tests and found suitable for parametric testing. Mixed Model Analysis of Variance tests (MM ANOVA) with post hoc pairwise comparisons was performed to assess the effects of two factors; the groups (older adults or younger adults) and 3 turning speeds (fast, moderate or slow). If significant interactions were seen between the two factors Repeated Measures Analysis of Variance tests (RM ANOVA) were performed to determine if differences exist between the 3 turning speeds within the 2 groups. Partial eta squared (η_p_^2^) was used to represent the effect size. Statistical significance was set at *p* < 0.05, and a Bonferroni correction was used for multiple comparisons. In addition, regression analyses between peak head yaw velocity and peak head-thorax and peak head-pelvis angular separation were used to assess intersegmental coordination.

## 3. Results

Fifty individuals (24 from younger adults and 26 from older adults) in total who lived independently were recruited from the local community of Salaya, Nakorn Pathom, Thailand. However, eight individuals (three from the younger adults and five from the older adults) did not meet the criteria, therefore, 42 participants in total (21 participants for each group) were included in the analysis. The 21 healthy older adults (OLD group) consisted of 9 males and 12 females, aged 66.4 ± 3.25 years, a mass of 60.98 ± 11.85 kg, and height of 1.59 ± 0.08 m.; and the 21 healthy younger adults (YOUNG group) consisted of 12 males and 9 females, aged 22.47 ± 2.18 years, a mass of 61.56 ± 11.18 kg, and height of 162.53 ± 10.64 cm.

### 3.1. Segment Onset Latencies

**Figure 3:**
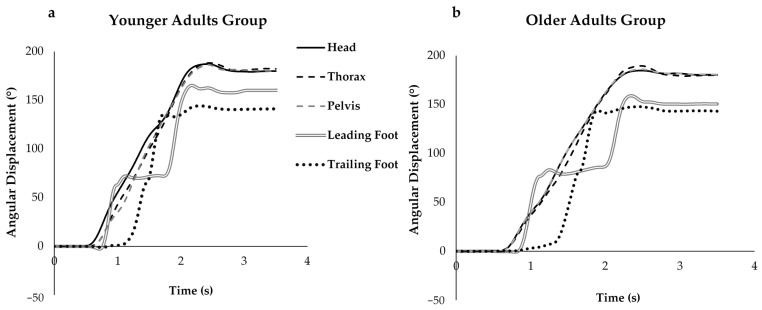
Turn displacement raw data from one trial of moderate speed in (**a**) younger adults group and (**b**) older adults group. This shows that segment reorientation began with the rotation of the head, followed by the trunk, pelvis, and the leading and trailing foot, which was seen in both groups.

Segment reorientation began with the head followed by the rotation of the trunk and pelvis, then the lead and trail feet; this sequence was preserved for each turning speed in both groups (Figure 3).

The MM ANOVA revealed significant interactions (*p* < 0.05) between groups and turn speeds for onset latency for the head, pelvis, leading foot, and trailing foot (Table 1). Therefore, the effects of turning speed within the two groups were further explored using RM ANOVA tests for these parameters.

**Figure 4:**
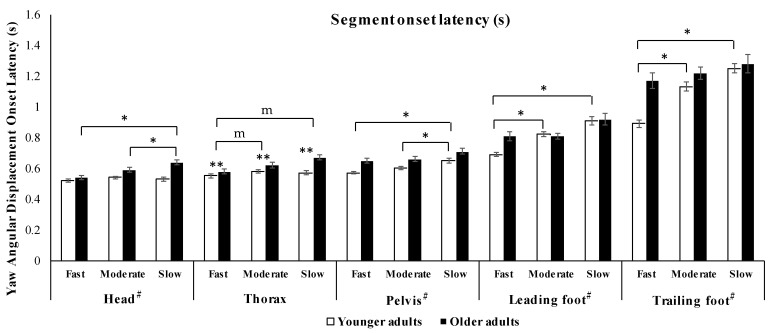
Bar graph showing the mean onset latencies with turn speed. There was a significant main effect of turn speed on the timing of reorientation onset for all segments. Values are mean ± SEM. # Indicates a significant interaction (*p* < 0.05) from MM ANOVA. * Indicates post-hoc pairwise comparisons of turning speeds within-group from RM ANOVA. ** Indicate main effects of group and turning speed from MM ANOVA. m Indicates post-hoc pairwise with a Bonferroni adjustment for multiple comparisons from MM ANOVA.

For head onset latency the RM ANOVA revealed a main effect of turning speed on (F_(2, 40)_ = 15.27, *p* = 0.031, η_p_^2^ = 0.16) within the OLD group only. Post-hoc pairwise comparisons showed that there was a significant decrease in the head onset latency between slow and fast speeds and slow and moderate speeds (Table 2 and Table 3 and Figure 4). Whereas, for pelvis onset latency, the RM ANOVA revealed a main effect of turning speed within the YOUNG group only (F_(2, 40)_ = 6.84, *p* < 0.05, η_p_^2^ = 0.031), with the post-hoc pairwise comparisons showing a significant increase between slow and fast speeds and slow and moderate speeds (Table 2 and Table 3).

For the leading foot onset latency, the RM ANOVA showed a main effect of turning speed only in the YOUNG group (F_(2, 40)_ = 18.62, *p* = 0.004, η_p_^2^ = 0.24). Post-hoc pairwise comparisons showed that the leading foot onset latency decreased significantly between moderate and fast speeds and slow and fast speeds (Table 3 and Table 4 and Figure 4). In addition, for the trailing foot onset latency, the RM ANOVA also found the main effect of turning speed within the YOUNG group only (F_(2, 40)_ = 24.07, *p* = 0.001, η_p_^2^ = 0.3). As with the leading foot onset latency, the post-hoc pairwise comparisons showed that the trailing foot onset latency also decreased significantly (*p* < 0.05) between moderate and fast speeds and slow and fast speeds (Table 2 and Table 3 and Figure 4).

The MM ANOVA showed no interactions between groups and turn speeds for the thorax onset latency. However, significant main effects were seen for the group (*p* < 0.001) and turn speed (*p* = 0.001) (Table 1). Post-hoc pairwise comparisons with a Bonferroni adjustment for multiple comparisons showed significant differences in thorax onset latency between slow and fast speeds and moderate and fast speeds (Table 4 and Figure 4). In addition, a significant difference between groups (*p* < 0.001) was seen in the MM ANOVA on thorax onset latency.

Interestingly, our results found that segment reorientation onset latency was shortest during fast speed trials and longest during slow speed trials for all segment onset latencies (Figure 4).

### 3.2. Intersegmental Coordination

**Figure 5:**
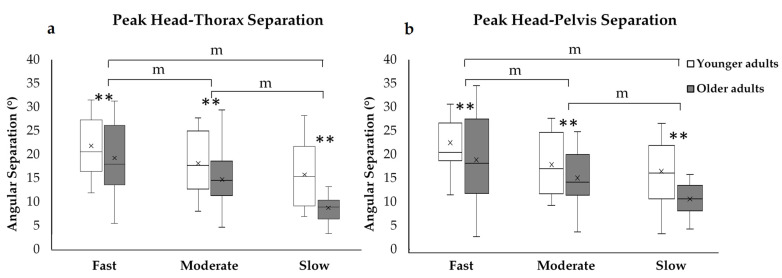
The effects of turning speed on mean (**a**) peak head–thorax angular separation and (**b**) peak head–pelvis angular separation under both conditions. The box and whisker plots illustrate the median peak head–thorax angular separation and peak head–pelvis angular separation. ** Indicate main effects of group and turn speed from MM ANOVA. m Indicates post-hoc pairwise with a Bonferroni adjustment for multiple comparisons from MM ANOVA.

The MM ANOVA revealed that there were no significant interactions between groups and turn speeds for intersegmental coordination. However, there was a significant main effect for both groups (*p* < 0.001) and turning speed (*p* < 0.001) on peak head-thorax and peak head-pelvis angular separations (Table 1). Further post-hoc pairwise comparisons with a Bonferroni adjustment for multiple comparisons found significant differences (*p* < 0.05) for peak head segmental angular separation between slow and fast speeds, slow and moderate speeds, and moderate and fast speeds, showing that peak segmental angular separation decreased with a decrease in turning speed (Table 4 and Figure 5). In addition, our results showed that the younger adults achieved a greater amount of peak head segmental angular separations than older adults.

When considering peak head yaw velocity, the MM ANOVA tests revealed significant interactions (*p* < 0.001) between groups and turn speeds (Table 1). Further RM ANOVA tests found a main effect of turning speed on the peak head yaw velocity in the OLD group (F_(2, 40)_ = 10.18, *p* = 0.001, η_p_^2^ = 0.41) and the YOUNG group (F_(2, 40)_ = 90.47, *p* < 0.0001, η_p_^2^ = 0.44) (Table 2). Post-hoc pairwise comparisons showed that the peak head yaw velocity decreased with a decrease in turn speed (*p* < 0.05) between slow and fast speeds, slow and moderate speeds, and moderate and fast speeds in the YOUNG group, whereas the OLD group only showed differences between the slow and fast speeds (Table 3).

**Figure 6:**
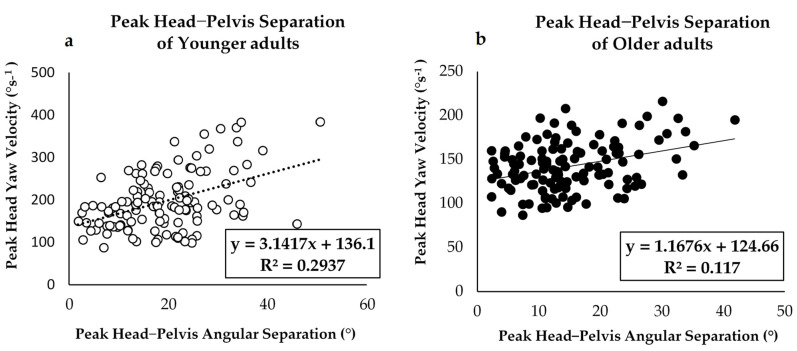
Scatterplot showing the results of regression analyses between peak head yaw velocity and maximum head-pelvis angular separation in (**a**) younger adults group and (**b**) older adults group, a significant positive correlation between peak head yaw velocity and the head-pelvis separation was found between groups (*p* < 0.05).

A further regression analysis between peak head yaw velocity and peak head–pelvis angular segment separation revealed a lower correlation in the OLD group when compared to the YOUNG group. However, both groups showed the existence of relationships between the head and pelvis under the turning speed condition, which predicts that turns performed above peak head velocities of approximately 100°s^−1^ will result in a separation between the head and pelvis during the turn and that the peak-pelvis angular separation increases with increasing turn speed (Figure 6).

### 3.3. Stepping Characteristics

**Figure 7:**
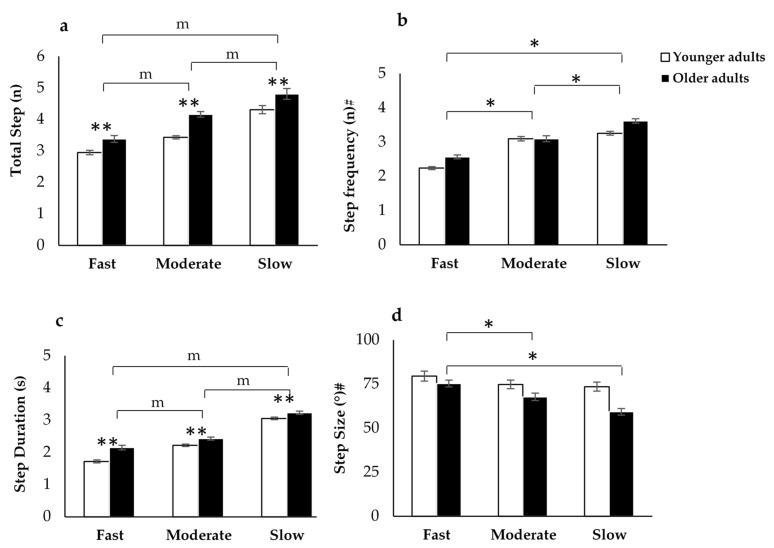
The effect of turn speed on (**a**) total step counts, (**b**) step frequency and (**c**) step duration taken to turn and (**d**) step size. Values are mean ± SEM. # Indicates a significant interaction (*p* < 0.05) from MM ANOVA. * Indicates post-hoc pairwise comparisons of turning speeds within group from RM ANOVA. ** Indicate main effects of group and speed from MM ANOVA. m Indicates post-hoc pairwise with a Bonferroni adjustment for multiple comparisons from MM ANOVA.

The comparison of total step, step frequency, step duration, and step size between groups and during turn speed conditions are presented in Table 1, Table 2, Table 3 and Table 4 and Figure 7. The MM ANOVA tests revealed no interaction between groups and turn speed for total step count, however, significant main effects were seen for groups (*p* < 0.001) and turn speed (*p* < 0.001) (Table 1). Post-hoc pairwise comparisons with a Bonferroni adjustment for multiple comparisons found significant differences (*p* < 0.001) in total step count between slow and fast speeds, moderate and fast speeds, and slow and moderate speeds, which showed that the number of steps was significantly greater during the slower turn speed (Table 4 and Figure 7). As with the total step count, for the step duration, the MM ANOVA tests revealed no interactions between groups and turn speed, however, significant main effects were seen for the group (*p* < 0.001) and speed (*p* < 0.001) (Table 1). Further post-hoc pairwise comparisons with a Bonferroni adjustment for multiple comparisons found significant differences (*p* < 0.05) between slow and fast speeds, slow and moderate speeds, and moderate and fast speeds, which showed that step duration was significantly greater during the slower turn speed (Table 4 and Figure 7).

For step frequency, a significant interaction between groups and turn speeds was found (*p* = 0.019) (Table 1). A further RM ANOVA test revealed a main effect of turning speed on step frequency within the OLD group (F_(2, 40)_ = 71.38, *p* < 0.001, η_p_^2^ = 0.62) and in the YOUNG group (F_(2, 40)_ = 55.67, *p* < 0.001, η_p_^2^ = 0.33) (Table 2). Post-hoc pairwise comparisons showed that the step frequency increased significantly (*p* < 0.001) with all decreases in turning speeds in both groups (Table 3 and Figure 7). As with step frequency, step size also showed an interaction between groups and turn speed (Table 1). A further RM ANOVA revealed a main effect of turning speed in only the OLD group (F_(2, 40)_ = 29.07, *p* < 0.001, η_p_^2^ = 0.47) (Table 2). In addition, post-hoc pairwise comparisons showed that significantly smaller step sizes (*p* < 0.001) were taken with the slower turning speed (Table 3 and Figure 7).

## 4. Discussion

The purpose of this study was to explore the effects of turn speed in healthy older adults compared to healthy younger adults and to observe its effects on body coordination and stepping characteristics. We hypothesized that turning at different speeds would result in changes in whole-body coordination and stepping behavior characteristics in older adults. We have accepted our hypotheses as the data shows that turning speed resulted in statistically significant changes in these characteristics not only in the older adults but also in the younger adults in some variables.

### 4.1. Segment Onset Latency

Previous studies have shown a clear top-down sequence of the onset of body segment reorientation during turning [5,8,19,20]. The horizontal movement starts with the eyes, which shift its gaze towards the new direction of travel; this is followed by head, trunk, pelvis yaws, and reorientation of the feet [4,7,15]. Our results are consistent with previous studies that used a similar methodology [15,18]. This current study found that there was an interaction effect between group and turn speed conditions on mean onset latency for all segments. When considering the onset latency of all segments at the three turn speeds, we found that the faster the turn speed, the earlier the rotation onset, showing the same temporal sequence between the younger and older adults groups. Interestingly, the older adults group had a longer onset latency than the younger adults group. This is consistent with the results of a previous study [10], indicating that the older adults may be responding to the differences in turn speed differently from the younger adults. It seems that the relative timing sequence is the same for each turn speed but is initiated sooner for faster turns, and these were constant between turns at each speed. This was consistent with the findings of previous studies [7], as well as segmental onset latency literature, which suggests that segmental onset latency may be controlled by a central nervous system (CNS) synergy [7]. This supports the notion that the various body segments are not controlled independently by the CNS, but rather are programmed as a sequence released earlier or later depending on the required speed of the turning movement [5,7]. This motor pattern can be adapted to control similar motor tasks, thus reducing the complexity of motor planning and reducing the reliance on sensory feedback [5,6]. Reed-Jones et al. in 2009 also found that a specific motor synergy task can be used by the CNS to control the reorganization of axial segments and to maintain dynamic balance and ongoing forward motion [6,7]. The controlling of the segmental timing sequence of the head, trunk, and feet redirection in each turn speed in the current study reflects the use of motor patterns to control dynamic postural reorientation, releasing the whole command from the brain to begin the turning sequence and produce movement [5,6]. This highly coordinated sequence could be used to control the ongoing trajectory of the lower segments during turning [15]. En-bloc turning has previously been documented in older adults and individuals with PD [11,21]. The previous study also found that the older adults initiated body segment rotation simultaneously during 360° turns [12,22]. Our results also support the previous study, which suggested that en-bloc segmental reorientation pattern may be adopted to simplify control turning movement pattern and may be an indicator of compensation for decreased postural stability and balance in frail populations during turning [22].

### 4.2. Peak Head Velocity and Peak Segmental Angular Separation Relationship

Traditionally, en-bloc turning, which is a strategy of altered turning behavior that puts older adults and individuals with PD at a greater risk of falling, has been characterized by reduced relative rotations between adjacent segments and near-simultaneous rotation initiation [21,22,23]. In this study, we found a significant difference (*p* < 0.001) between peak head velocity, the peak head-thorax, and the peak head-pelvis angular separation between groups. These results are consistent with the findings of Forsell et al. [23]. Our results (Figure 6) suggest that when turning at speeds less than 100°s^−1^ the head and body move in an en-bloc pattern, whereas at faster speeds, the head leads the pelvis by as much as 40–50° in healthy younger adults and only 20–30° in older adults. Despite this evidence that participants turn with a slow peak head yaw speed, their intersegmental coordination was interrupted [15,22]. A fixed characteristic or en-bloc appearance was observed during turns along with smaller reciprocal movements between either the head and thorax or the head and pelvis. These may be related to a shorter stride length and/or slower gait speed, as either would diminish the need for counterbalancing pelvic rotation [22,23,24]. Alternatively, this might result from joint stiffness in older adults. Participants who turned with a slow speed and restrained their heads might not have retained a reciprocal oscillating pattern during turns [23]. Overall, it is worth noting that slow turning in older adults results in difficulty while performing daily activities, especially those that require turning or sequential movements and leads to an increased risk of falling [22,23,24,25].

### 4.3. Stepping Characteristics

We found that the foot rotation during the swing phase (step size) reduced, whereas the total number of steps, step duration, and step frequency increased during slower turns in older adults compared to younger adults. Our results suggest that small, frequent steps may also be partially explained by a generalized effect of simply moving slowly. It is generally agreed upon that turning step characteristics are frequently used to measure turning difficulties in older adults, as well as in individuals with neurological conditions, especially individuals with PD [17,21,25,26]. These findings support the hypothesis that turning speed and stepping behavior are intrinsically linked in interactive fall prevention [19,20]. Older adults take extra turning time, number of steps, and make wider turns with small steps to increase stability during turning [3,19,20,27]. It has been indicated that the presence of characteristics of turning disturbances increases the risk of falling. According to Akram et al. [8], older adults who have impaired gait stability take extra turning time and turning steps to compensate for lack of stability [20]. This finding suggests that stepping characteristics in older adults may be the direct result of an intentionally slow turning strategy to compensate for perceived or actual instability.

There are several limitations to this study. Sex differences were not included in the selected characteristics. It would have been useful to compare the older group of adults to a sex-matched group of younger healthy adults, or an age-matched group of adults with no mobility issues, or observe the differences of whole-body coordination during turning between the early older adults and the oldest adults. This would give important information regarding the extent of turning deficits in this population. A second limitation was that we did not include eye movement in the analysis. In future work, it is recommended that this is included to investigate any link with whole-body coordination, balance, and posture during on-the-spot turns and walking turns. This would further our understanding of the mechanisms that underlie turning problems and the risk of falls in older adults. To relate the findings of this study to problems associated with the risk of falling in daily life, a questionnaire could be used before and after the experiment. This would enable the exploration of which aspects of home life are of higher risk and the recommendation of appropriate precautions.

## 5. Conclusions

This study demonstrates systematic relationships between turning speed on whole-body coordination during standing turns. Our results indicate that turning speeds result in altered whole-body coordination and stepping behavior in older adults with the same temporal–spatial sequence as younger adults. However, some characteristics differ significantly, e.g., onset latency of segments, peak head velocity, step frequency, and step size. Importantly, the extent to which a turn is carried out using intersegmental coordination is dependent on the turning speed. These strategies may assist in the maintenance of balance while changing the turn speed in the presence of age-related physiological deficits and/or low balance confidence. Thus, determining and quantifying the turning movement dysfunction related to this fall-provoking activity may be useful for identifying individuals who are at risk of falling, which may be used to guide more effective training during turning in older individuals.

## Figures and Tables

**Table 1:** Mean and standard deviations (SD) and interaction between group and turning speed for whole-body coordination and stepping characteristics variables which performed by MM ANOVA.

Variables	OLD Group (n = 21)			YOUNG Group (n = 21)				
	Slow	Moderate	Fast	Slow	Moderate	Fast	Group Effect *p*-Value (η_p_^2^)	Speed Effect *p*-Value (η_p_^2^)
**Head onset (s)** **#**	0.63 (0.07)	0.59 (0.04)	0.54 (0.06)	0.53 (0.08)	0.54 (0.07)	0.52 (0.05)	<0.001 * (0.18)	0.001 * (0.16)
**Thorax onset (s)**	0.67 (0.07)	0.62 (0.06)	0.58 (0.07)	0.57 (0.07)	0.58 (0.08)	0.55 (0.06)	<0.001 * (0.15)	0.001 * (0.16)
**Pelvis onset (s) #**	0.71 (0.09)	0.68 (0.06)	0.65 (0.09)	0.65 (0.06)	0.60 (0.07)	0.57 (0.04)	0.003 * (0.11)	0.001 * (0.14)
**Leading foot onset (s) #**	0.92 (0.13)	0.81 (0.08)	0.81 (0.13)	0.91 (0.18)	0.82 (0.10)	0.69 (0.08)	0.051 (0.03)	<0.001 * (0.27)
**Trailing foot onset (s) #**	1.28 (0.14)	1.22 (0.21)	1.17 (0.21)	1.25 (0.27)	1.13 (0.15)	0.89 (0.11)	<0.001 * (0.12)	<0.001 * (0.28)
**Peak head yaw velocity (°s^−1^) #**	128.17(15.17)	141.32 (23.35)	155.45 (24.08)	136.75 (24.18)	181.45 (19.60)	271.53 (48.23)	<0.001 * (0.59)	<0.001 * (0.71)
**Peak head-thorax angular separation (°)**	9.45 (3.33)	14.67 (5.23)	18.70 (7.56)	16.53 (7.19)	17.63 (5.99)	21.75 (7.70)	<0.001 * (0.12)	<0.001 * (0.26)
**Peak head-pelvis angular separation (°)**	9.89 (3.61)	15.74 (5.93)	19.08 (8.60)	15.70 (6.85)	18.49 (5.99)	22.46 (6.61)	0.001 * (0.1)	<0.001 * (0.31)
**Total step (n)**	4.81 (0.80)	4.16 (0.24)	3.38 (0.49)	4.31 (0.60)	3.43 (0.40)	2.95 (0.31)	<0.001 * (0.31)	<0.001 * (0.59)
**Step frequency** **(n) #**	3.62 (0.30)	3.10 (0.30)	2.57 (0.21)	3.26 (0.24)	3.10 (0.40)	2.24 (0.26	<0.001 * (0.16)	<0.001 * (0.18)
**Step duration (s)**	3.23 (0.18)	2.43 (0.17)	2.15 (0.32)	3.05 (0.26)	2.22 (0.22)	1.72 (0.21)	<0.001 * (0.27)	<0.001 * (0.88)
**Step size (°) #**	59.21 (8.57)	67.68 (9.65)	75.22 (8.91)	73.47 (11.54)	74.71 (11.16)	79.46 (12.91)	<0.001 * (0.15)	<0.001 * (0.23)

# Indicates a significant interaction (*p* < 0.05). * Indicates significant main effects (*p* < 0.05). MM ANOVA = Mixed Model Analysis of Variance.

**Table 2:** RM ANOVA for variables that showed a significant interaction within MM ANOVA.

Groups	Variables	Speeds			Speed Effect *p*-Value (η_p_^2^)
		Slow	Moderate	Fast	
**Old Group**	Head onset (s)	0.63 (0.07)	0.59 (0.04)	0.54 (0.06)	0.031 * (0.16)
	Pelvis onset (s)	0.71 (0.09)	0.68 (0.06)	0.65 (0.09)	0.194 (0.08)
	Leading foot onset (s)	0.92 (0.13)	0.81 (0.08)	0.81 (0.13)	0.125 (0.10)
	Trailing foot onset (s)	1.28 (0.14)	1.22 (0.21)	1.17 (0.21)	0.411 (0.04)
	Peak head yaw velocity (°s^−1^)	128.17 (15.17)	141.32 (23.35)	155.45 (24.08)	0.001 * (0.41)
	Step frequency (n)	3.62 (0.30)	3.10 (0.30)	2.57 (0.21)	<0.001 * (0.62)
	Step size (°)	59.21 (8.57)	67.68 (9.65)	75.22 (8.91)	<0.001 * (0.47)
**Young Group**	Head onset (s)	0.53 (0.08)	0.53 (0.08)	0.53 (0.08)	0.775 (0.01)
	Pelvis onset (s)	0.65 (0.06)	0.60 (0.07)	0.57(0.04)	<0.001 * (0.32)
	Leading foot onset (s)	0.91 (0.18)	0.82 (0.10)	0.69 (0.08)	0.004 * (0.24)
	Trailing foot onset (s)	1.25 (0.27)	1.13 (0.15)	0.89 (0.11)	0.001 * (0.3)
	Peak head yaw velocity (°s^−1^)	136.75 (24.18)	181.45 (19.60)	271.53 (48.23)	<0.001 * (0.44)
	Step frequency (n)	3.26 (0.24)	3.10 (0.40)	2.24 (0.26	<0.001 * (0.33)
	Step size (°)	73.47 (11.54)	74.71 (11.16)	79.46 (12.91)	0.518 (0.03)

* Indicates a significant main effect (*p* < 0.05). RM ANOVA = Repeated Measures Analysis of Variance. MM ANOVA = Mixed Model Analysis of Variance. OLD = older adults group, and YOUNG = younger adults group.

**Table 3:** Post hoc comparisons RM ANOVA revealed the effects of turning speed in each group.

Groups	Variables	Speeds	Mean Diff (SE)	*p*-Value	CI of Diffs	
					Lower Bound	Upper Bound
**OLD group**	Head onset (s)	Slow to Fast	0.10 (0.02)	<0.001 *	0.052	0.150
		Slow to Moderate	0.05 (0.02)	0.019 *	0.007	0.094
		Moderate to Fast	0.05 (0.02)	0.052	0.000	0.101
	Peak head yaw velocity (°s^−1^)	Slow to Fast	−27.25 (6.27)	0.001 *	−43.657	−10.909
		Slow to Moderate	−13.15 (6.40)	0.160	−29.878	3.579
		Moderate to Fast	−14.13 (5.43)	0.051	−28.307	0.039
	Step frequency (n)	Slow to Fast	1.05 (0.06)	<0.001 *	0.889	1.220
		Slow to Moderate	0.52 (0.10)	<0.001 *	0.246	0.795
		Moderate to Fast	0.53 (0.10)	<0.001 *	0.295	0.772
	Step size (°)	Slow to Fast	−16.01 (2.05)	<0.001 *	−21.372	−10.655
		Slow to Moderate	−8.47 (2.27)	0.004 *	−14.399	−2.539
		Moderate to Fast	−7.54 (1.97)	0.003 *	−12.696	−2.392
**YOUNG group**	Pelvis onset (s)	Slow to Fast	0.08 (0.02)	0.018 *	0.010	0.119
		Slow to Moderate	0.05 (0.02)	0.002 *	−0.017	0.035
		Moderate to Fast	0.03 (0.01)	0.053	0.028	0.123
	Leading foot onset (s)	Slow to Fast	0.22 (0.04)	<0.001 *	0.125	0.319
		Slow to Moderate	0.09 (0.04)	0.190	−0.028	0.197
		Moderate to Fast	0.14 (0.03)	<0.001 *	0.063	0.211
	Trailing foot onset (s)	Slow to Fast	0.37 (0.05)	<0.001 *	0.225	0.506
		Slow to Moderate	0.13 (0.06)	0.179	−0.040	0.298
		Moderate to Fast	0.24 (0.04)	<0.001 *	0.136	0.336
	Peak head yaw velocity (°s^−1^)	Slow to Fast	−134.79 (11.93)	<0.001 *	−165.95	−103.62
		Slow to Moderate	−44.71 (7.70)	<0.001 *	−64.84	−24.57
		Moderate to Fast	−90.01 (10.53)	<0.001 *	−117.59	−62.57
	Step frequency (n)	Slow to Fast	1.02 (0.07)	<0.001 *	0.832	1.199
		Slow to Moderate	0.152 (0.11)	0.546	−0.136	0.440
		Moderate to Fast	0.863 (0.12)	<0.001 *	0.540	1.186

* Indicates a significant difference (*p* < 0.05). RM ANOVA = Repeated Measures Analysis of Variance. Diff = Difference. CI = Confidence Intervals. OLD = older adults group, and YOUNG = younger adults group.

**Table 4:** Post hoc comparisons for the main effects seen in the MM ANOVA, where no interactions between group and turning speed were seen.

Variables	Both Groups Combined	Mean Diff (SE)	*p*-Value	CI of Diffs	
				Lower Bound	Upper Bound
**Thorax onset (s)**	Slow to Fast	0.06 (0.02)	0.001 *	0.022	0.094
	Slow to Moderate	0.02 (0.02)	0.456	−0.015	0.058
	Moderate to Fast	0.04 (0.01)	0.044 *	0.001	0.071
	YOUNG to OLD	−0.06 (0.01)	<0.001 *	−0.80	−0.33
**Peak head-thorax** **angular separation (°)**	Slow to Fast	−7.24 (1.46)	<0.001 *	−10.815	−3.657
	Slow to Moderate	−3.16 (1.23)	0.035 *	−6.157	−0.167
	Moderate to Fast	−4.07 (1.46)	0.020	−7.657	−0.491
	YOUNG to OLD	4.36 (1.13)	<0.001 *	2.118	6.117
**Peak head-pelvis** **angular separation (°)**	Slow to Fast	−7.98 (1.45)	<0.001 *	−11.54	−4.42
	Slow to Moderate	−4.32 (1.25)	0.003	−7.377	−1.268
	Moderate to Fast	−3.66 (1.50)	0.051	−7.327	0.013
	YOUNG to OLD	3.98 (1.15)	0.001 *	1.706	6.252
**Total step (n)**	Slow to Fast	1.40 (0.13)	<0.001 *	1.090	1.711
	Slow to Moderate	0.77 (0.12)	<0.001 *	0.473	1.065
	Moderate to Fast	0.63 (0.08)	<0.001 *	0.432	0.830
	YOUNG to OLD	−0.55 (0.09)	<0.001 *	−0.734	−0.374
**Step duration (s)**	Slow to Fast	1.21 (0.05)	<0.001 *	1.072	1.339
	Slow to Moderate	0.81 (0.05)	<0.001 *	0.700	0.925
	Moderate to Fast	0.39 (0.05)	<0.001 *	0.267	0.519
	YOUNG to OLD	−0.27 (0.04)	<0.001 *	−0.350	−0.186

* Indicates a significant difference (*p* < 0.05), MM ANOVA = Mixed Model Analysis of Variance, Diff = Difference, CI = Confidence Intervals, OLD = older adults group, and YOUNG = younger adults group.

## Data Availability

The data presented in this study are available on request from the corresponding author.
